# Immobilization of β-Agarase onto Ni-NTA Magnetic Nanoparticles for Efficient Production of Functional Neoagarooligosaccharides

**DOI:** 10.3390/foods15162778

**Published:** 2026-08-07

**Authors:** Kaifan Qiu, Chen Wang, Xingfei Li, Zhengyu Jin, Jie Long

**Affiliations:** 1 The State Key Laboratory of Food Science and Technology, Jiangnan University, 1800 Lihu Road, Wuxi 214122, China; 6240111223@stu.jiangnan.edu.cn (K.Q.); 6220111224@stu.jiangnan.edu.cn (C.W.); 8201901026@jiangnan.edu.cn (X.L.); fpcenter@jiangnan.edu.cn (Z.J.); 2 School of Food Science and Technology, Jiangnan University, 1800 Lihu Road, Wuxi 214122, China; 3 Collaborative Innovation Center of Food Safety and Quality Control in Jiangsu Province, Jiangnan University, Wuxi 214122, China

**Keywords:** β-agarase, enzyme immobilization, Ni-NTA magnetic nanoparticles, neoagarooligosaccharides, functional oligosaccharides, agarose hydrolysis

## Abstract

AgaDcat is a promising β-agarase for the production of functional neoagarooligosaccharides (NAOSs), but its limited thermal stability and poor reusability as a free enzyme increase enzyme consumption and production cost during repeated agarose hydrolysis. In this study, β-agarase AgaDcat was immobilized on nickel-nitrilotriacetic acid magnetic nanoparticles (Ni-NTA-MNPs) through His-tag/Ni-NTA affinity interactions. The successful preparation of Ni-NTA-MNPs and the immobilization of AgaDcat were verified by SEM, EDS, VSM, FT-IR, XRD, and TGA. Following optimization, the immobilized enzyme exhibited an activity retention of 80.47%. Thermal stability assays showed that the immobilized enzyme was more thermostable than the free enzyme. After incubation at 50 °C for 60 min, the immobilized enzyme retained 59.4% of its initial activity, whereas the free enzyme retained only 29.1%. Moreover, the immobilized enzyme displayed good reusability, retaining 90.59% and 58.71% of its activity after 3 and 7 cycles, respectively. HPAEC-PAD analysis showed that the immobilized enzyme reached reaction equilibrium within 4–8 h, with neoagarotetraose (NA4) and neoagarohexaose (NA6) identified as the major degradation products, whereas the free enzyme required 20 h, indicating significantly improved catalytic efficiency. These results indicate that IMAC-based immobilization improves the stability, reusability, and catalytic efficiency of β-agarase, providing a promising reusable biocatalytic strategy for the efficient production of functional neoagarooligosaccharides.

## 1. Introduction

Enzyme immobilization is widely used to improve enzyme stability, reusability, and operational convenience in biocatalytic processes. For food-related oligosaccharide production, immobilized enzymes are particularly attractive because they can reduce enzyme consumption, simplify catalyst recovery, and facilitate repeated-batch processing. In some cases, immobilization can also enhance enzyme tolerance to unfavorable reaction conditions, thereby improving the feasibility of enzymatic production of functional ingredients [1]. Common enzyme immobilization methods include physical adsorption, covalent binding, and entrapment [2]. Magnetic nanoparticles have been extensively studied for enzyme immobilization owing to their abundant surface active sites, facile multifunctional modification, excellent biocompatibility, and convenient magnetic-field-driven separation. Various methods for the synthesis of magnetic nanomaterials have been developed over the years, including co-precipitation [3], aerosol routes [4], hydrothermal reactions [5], organic precursor methods [6], and sol–gel synthesis [7].

Previous studies on β-agarase immobilization have mainly used nonspecific adsorption, covalent binding, or bioaffinity-based strategies, such as tannic acid-modified Fe_3_O_4_ nanoparticles [8], biotin/streptavidin-modified Fe_3_O_4_ nanoparticles [9], and polydopamine-based magnetic carriers [10]. Although these approaches can improve enzyme recovery and reuse, they may still suffer from activity loss caused by random enzyme attachment, steric hindrance, mass-transfer limitation, or enzyme detachment during repeated operation. Therefore, developing an immobilization strategy that combines mild immobilization conditions, efficient enzyme loading, stable attachment, and convenient magnetic recovery remains important for reusable β-agarase catalysis.

Immobilized metal-ion affinity chromatography (IMAC) provides a useful route for immobilizing polyhistidine-tagged proteins through the coordination between histidine residues and metal-chelating ligands on solid supports. In this system, chelating ligands such as iminodiacetic acid (IDA), nitrilotriacetic acid (NTA), carboxymethylaspartic acid (CM-Asp), and tris(carboxymethyl)ethylenediamine (TED) can coordinate metal ions such as Ni^2+^, which subsequently bind His-tagged proteins under mild conditions [11,12,13,14]. Compared with random covalent immobilization, His-tag/Ni-NTA-mediated immobilization mainly relies on affinity interaction between the terminal His tag and Ni-NTA groups, which may reduce nonspecific modification of the enzyme surface and help preserve catalytic activity. In addition, magnetic nanoparticles can be rapidly recovered from the reaction system using an external magnetic field, thereby reducing enzyme loss during repeated-batch operation. However, the application of Ni-NTA magnetic nanoparticles for immobilizing His-tagged β-agarase and producing functional neoagarooligosaccharides has not been sufficiently investigated.

Agarase is typically isolated from marine bacteria involved in red algae decomposition. Due to its marine origin, agarase operates optimally at a weakly alkaline pH (7–9) and moderate temperatures (30–50 °C) but exhibits poor heat resistance. Agarases are classified based on their cleavage site on agarose chains into α-agarase (EC 3.2.1.158) and β-agarase (EC 3.2.1.81) [15]. AgaDcat is a catalytic domain of AgaD from *Zobellia galactanivorans DsiJT*, identified by Hehemann et al. [16] (PDB: 4ASM). As an endonuclease in GH16, AgaDcat cleaves β-1,4-glycosidic bonds to produce neoagarooligosaccharides (NAOS) terminated with 3,6-anhydro-L-galactose (L-AHG). NAOS exhibit various physiological activities including antioxidant, anti-inflammatory, hepatoprotective, probiotic, anti-tumor, and melanogenesis inhibition effects. Different degrees of polymerization in NAOS result in distinct physiological activities; for instance, neoagarobiose (NA2) improves male pattern baldness induced by dihydrotestosterone (DHT) [17], neoagarotetraose (NA4) alleviates exercise-induced fatigue stress in mice by normalizing blood glucose and lactate levels [18], and neoagarohexaose (NA6) demonstrates significant antitumor effects on B16F1 melanoma cells [19].

**Figure 1:**
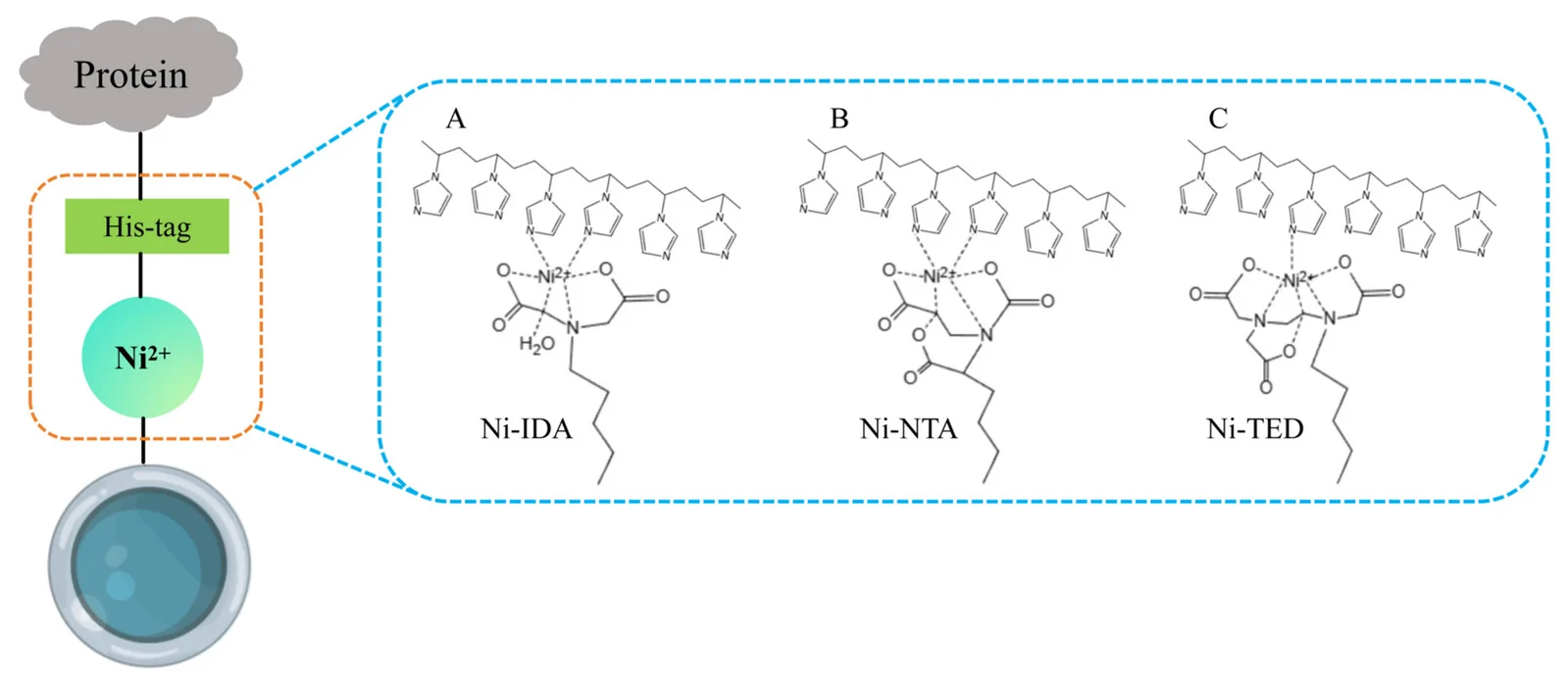
Schematic illustration of His-tag/Ni^2+^ affinity-mediated immobilization on magnetic nanoparticles. The His-tagged protein coordinates with Ni^2+^ chelated by metal-affinity ligands on the magnetic nanoparticle surface. The inset shows representative Ni^2+^-chelating ligands, including Ni-IDA, Ni-NTA, and Ni-TED, with Ni-NTA used in the present study.

Beyond the well-characterized functions of individual NAOS components, the binary mixture of NA4 and NA6 exhibits more comprehensive and application-oriented bioactivities that have been widely verified in metabolic regulation research. This mixture exerts prominent anti-obesity, anti-diabetic, and hypolipidemic effects by modulating key lipid metabolism signaling pathways, improving insulin sensitivity, and inhibiting abnormal lipid accumulation in serum and adipose tissues. Meanwhile, it can alleviate liver function damage and hepatic steatosis, showing favorable hepatoprotective activity, which makes it a high-value active ingredient for functional foods and dietary supplements [20,21]. AgaDcat shows high catalytic activity and can efficiently hydrolyze agarose into NA4 and NA6 as the main products, together with a small amount of neoagarooctaose (NA8), thus representing a highly promising candidate for industrial production of functional neoagaro-oligosaccharides. Therefore, the efficient preparation of NAOS, especially NA4 and NA6, is of considerable interest for the development of marine polysaccharide-derived functional ingredients. However, practical enzymatic production is still limited by the poor thermostability of agarases, enzyme loss during repeated use, and the relatively high cost of soluble enzymes. Developing a reusable and easily recoverable agarase biocatalyst is therefore important for improving the feasibility of NAOS production in food-related applications. The principle of His-tag/Ni^2+^ affinity-based enzyme immobilization is illustrated in Figure 1. In this strategy, the His-tag of AgaDcat coordinates with Ni^2+^ chelated by NTA groups on the surface of magnetic nanoparticles, allowing affinity-mediated immobilization under mild conditions. Compared with random covalent immobilization, this strategy may reduce nonspecific modification of the enzyme surface and facilitate magnetic recovery of the immobilized biocatalyst.

Compared with previously reported β-agarase immobilization methods, such as nonspecific adsorption, random covalent attachment, and bioaffinity coupling, the IMAC-based Ni-NTA/His-tag strategy used in this study provides a more selective route for immobilizing recombinant β-agarase. In this system, the His-tag of AgaDcat specifically coordinates with Ni^2+^ chelated by NTA groups on the magnetic nanoparticles, allowing affinity-driven immobilization under mild conditions. This strategy can reduce nonspecific enzyme attachment, avoid extensive random modification of the enzyme surface, and minimize the risk of disturbing the catalytic center, thereby helping to preserve enzyme activity. Therefore, the use of Ni-NTA magnetic nanoparticles for His-tagged β-agarase represents an affinity-mediated and operationally convenient immobilization strategy for reusable neoagarooligosaccharide production.

In this work, an immobilized metal-ion affinity chromatography (IMAC)-based strategy was developed to immobilize His-tagged AgaDcat onto Ni-NTA-modified magnetic nanoparticles. This strategy enables the specific binding of AgaDcat through its His tag and allows rapid recovery of the immobilized enzyme by magnetic separation. The catalytic properties of free and immobilized AgaDcat were systematically compared in terms of immobilization efficiency, optimal reaction conditions, thermal stability, kinetic parameters, reusability, and hydrolysis product profiles. This study provides a reusable biocatalytic platform for the efficient production of functional neoagarooligosaccharides.

## 2. Materials and Methods

**Figure 2:**
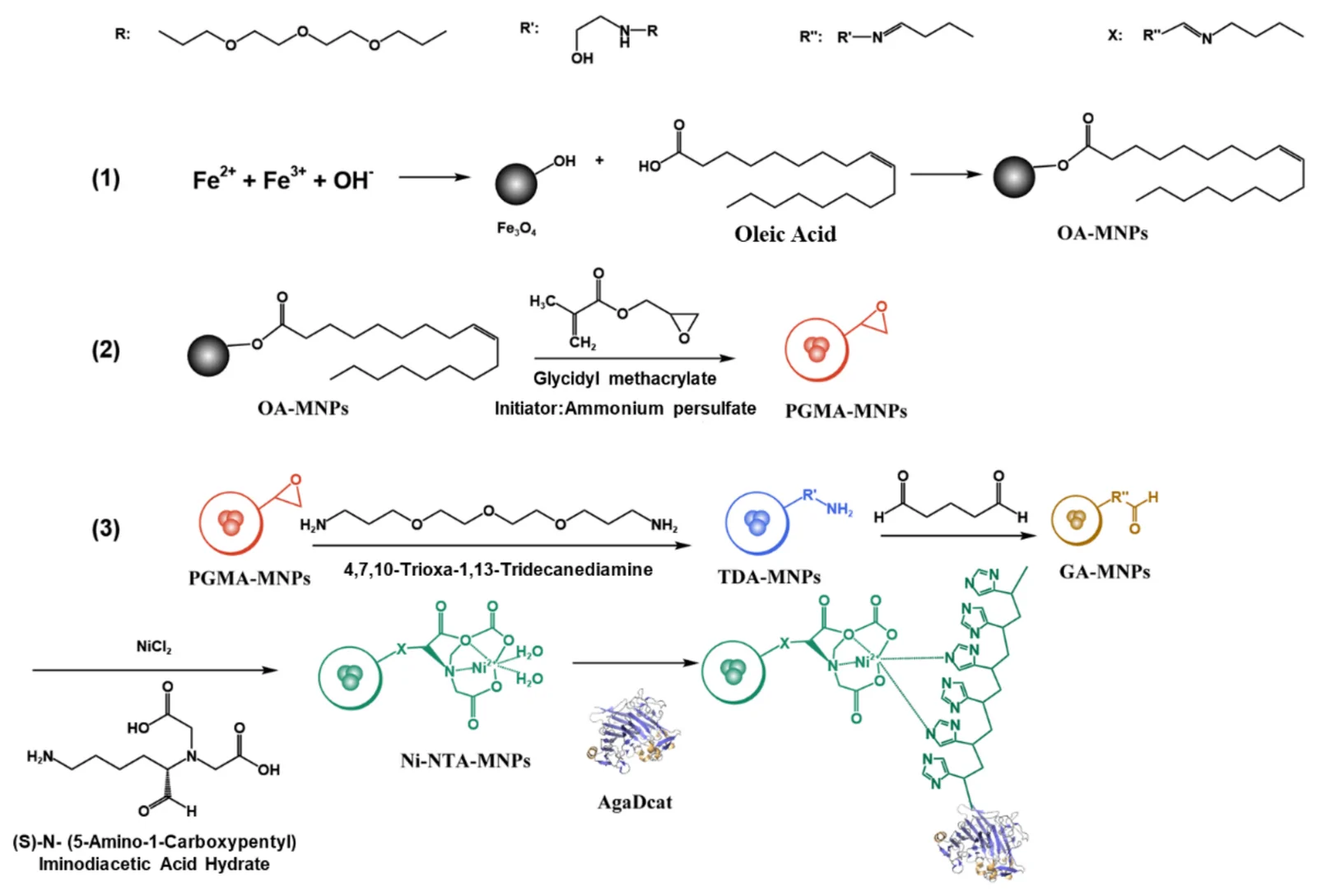
Representative scheme showing the chemical mechanism for the preparation and functionalization of Ni-NTA-MNPs and the subsequent immobilization of His-tagged AgaDcat. The scheme illustrates the formation of Fe_3_O_4_ magnetic nanoparticles, oleic acid modification, PGMA grafting, introduction of amino-containing spacer arms, glutaraldehyde-mediated support activation, introduction of NTA-like chelating groups, Ni^2+^ chelation, and His-tag/Ni-NTA-mediated immobilization of AgaDcat. Glutaraldehyde was used for support functionalization, whereas AgaDcat immobilization was mainly mediated by His-tag/Ni-NTA coordination rather than direct glutaraldehyde-mediated covalent crosslinking.

The overall experimental workflow for the preparation of Ni-NTA-MNPs and immobilization of His-tagged AgaDcat is shown in Figure 2.

### 2.1. Recombinant Expression and Purification of β-Agarase AgaDcat

The recombinant *E. coli* strain harboring the pET-28a(+)-AgaDcat plasmid was used for AgaDcat expression. The plasmid map of pET-28a(+)-AgaDcat is provided in Figure S1. AgaDcat corresponds to the catalytic module of full-length AgaD from *Zobellia galactanivorans* DsiJT. Previous biochemical and structural studies have shown that AgaD is a modular β-agarase, whose catalytic module has been successfully expressed, purified, crystallized, and biochemically characterized, with its crystal structure deposited as PDB ID 4ASM [16]. The isolated catalytic module retains β-agarase activity and can efficiently hydrolyze agarose to produce NA4 and NA6 as the main products [16]. Therefore, the use of AgaDcat in this study was based on a structurally characterized and functionally validated catalytic module rather than an arbitrary truncation of the full-length enzyme. Compared with the full-length enzyme, AgaDcat provides a smaller and more defined catalytic unit, which can reduce possible interference from non-catalytic regions and facilitate recombinant expression, purification, immobilization, and enzymatic characterization. LB liquid medium contained 10 g/L tryptone, 5 g/L yeast extract, and 10 g/L NaCl, while LB solid medium was prepared by adding 15 g/L agar. Tryptone and yeast extract were purchased from Oxoid Ltd., and NaCl and agar were purchased from Sinopharm Chemical Reagent Co., Ltd. (Shanghai, China). Kanamycin was added to a final concentration of 50 μg/mL. Isopropyl-β-D-thiogalactoside (IPTG) was obtained from Beijing Inokai Technology Co., Ltd. (Beijing, China). The recombinant strain was streaked onto LB solid medium and incubated at 37 °C until single colonies appeared. Single colonies were then inoculated into 5 mL of LB liquid medium and cultured overnight at 37 °C and 200 r/min to prepare the seed culture. The seed culture was transferred into 100 mL of fresh LB liquid medium at a 1% (*v*/*v*) inoculum ratio and incubated until the optical density at 600 nm (OD600) reached 0.6–0.8. IPTG was then added to a final concentration of 1 mM, and enzyme expression was induced at 16 °C and 150 r/min for 12 h. The cells were harvested by centrifugation at 8000 rpm for 10 min at 4 °C and disrupted on ice using an ultrasonic cell crusher at 40% output power with a pulse mode of 2 s on and 3 s off for a total sonication time of 20 min. After centrifugation at 8000 rpm for 10 min at 4 °C to remove cell debris, the supernatant was filtered through a 0.45 μm PES syringe filter purchased from Labfil/ALWSCI (Shaoxing, China) and purified using HisTrap™ affinity chromatography (Cytiva, Little Chalfont, UK). The purified AgaDcat yield was 60 mg/L culture. The specific activity increased from 139.68 U/mg-protein in the crude extract to 3057 U/mg-protein after purification, corresponding to a 21.89-fold increase in specific activity.

### 2.2. Preparation of Ni-NTA-MNPs Magnetic Nanoparticles

#### 2.2.1. Synthesis of OA-Modified Fe_3_O_4_ via Co-Precipitation Method

Ferric chloride hexahydrate, ferrous chloride tetrahydrate, oleic acid (OA), glutaraldehyde, nickel chloride, and potassium bromide were purchased from Sinopharm Chemical Reagent Co., Ltd. (Shanghai, China). Glycidyl methacrylate and 4,7,10-trioxo-1,13-tridecanediamine were obtained from Shanghai Macklin Biochemical Technology Co., Ltd. (Shanghai, China). N,N-bis(carboxymethyl)-L-lysine was purchased from Beijing Solarbio Technology Co., Ltd. (Beijing, China). These reagents were used in the stepwise preparation of OA-modified Fe_3_O_4_, PGMA-MNPs, TDA-MNPs, and Ni-NTA-MNPs as described below.

The synthesis of OA-modified magnetic nanoparticles followed the method described by Wang et al. [22], with minor modifications. Briefly, 9.8 g of FeCl_2_·4H_2_O and 24 g of FeCl_3_·6H_2_O were dissolved in 400 mL of ultrapure water and mechanically stirred at 700 r/min in an 80 °C water bath. After the addition of 25% ammonia solution, the precipitate rapidly turned black, indicating the formation of Fe_3_O_4_. The pH of the suspension was adjusted to 7.0 using hydrochloric acid, and the reaction was continued for 30 min at 80 °C under mechanical stirring. Subsequently, 3.0 mL of oleic acid was added to the reaction system, and the mixture was mechanically stirred for another 1.5 h at 80 °C to allow surface modification. No emulsifying agent was added during this process. The nanoparticles were then magnetically separated using an external magnet and washed with deionized water and anhydrous ethanol until neutral. Magnetic separation throughout this study was performed using a neodymium–iron–boron (NdFeB) permanent magnet with a surface magnetic flux density of approximately 300–500 mT. After washing, the OA-MNPs were collected by magnetic separation, dried at 60 °C to constant weight, and stored in a sealed container at room temperature (25 ± 2 °C) until further use.

#### 2.2.2. Synthesis of PGMA-MNPs

To synthesize PGMA-MNPs, 4.15 g of OA-MNPs were suspended in 400 mL of ultrapure water, followed by the addition of 0.25 g of ammonium persulfate and 3.16 mL of glycidyl methacrylate. The mixture was stirred at 80 °C for 6 h to obtain Glycidyl Methacrylate-modified MNPs (PGMA-MNPs). After the reaction, the PGMA-MNPs were magnetically separated using an external permanent magnet, washed several times with deionized water, dried in an oven at 60 °C to constant weight, and stored in a sealed container at room temperature (25 ± 2 °C) until further use.

#### 2.2.3. Synthesis of Ni-NTA-MNPs

The synthesis procedure for Ni-NTA-MNPs was adapted from Vahidi et al. [23]. First, 3 g of PGMA-MNPs were suspended in 300 mL of ultrapure water, and 30 mL of 13 mM 4,7,10-trioxo-1,13-tridecanediamine was added and stirred at 80 °C for 10 h to obtain TDA-MNPs. These were washed with deionized water three times and separated magnetically. The TDA-MNPs were then reacted with 300 mL of 25% glutaraldehyde aqueous solution at 80 °C for 3 h, followed by washing and resuspension in 200 mL of ultrapure water. Next, 30 mL of N,N-bis(carboxymethyl)-L-lysine (50 mM in potassium phosphate buffer, pH 8.0) was added dropwise at room temperature (25 ± 2 °C) with stirring for 12 h. After washing with deionized water three times, the nanoparticles were resuspended in 150 mL of ultrapure water. Finally, 5 mL of NiCl_2_ solution (1 M) was added dropwise and stirred for 2 h at room temperature (25 ± 2 °C). The obtained Ni-NTA-MNPs were magnetically separated using an external permanent magnet, washed three times with deionized water, dried in an oven at 60 °C to constant weight, and stored in a sealed container at room temperature (25 ± 2 °C) until use. The chemical mechanism involved in the preparation and functionalization of Ni-NTA-MNPs is summarized in Figure 2. Briefly, PGMA was first grafted onto the surface of Fe_3_O_4_ magnetic nanoparticles, and the epoxy groups of PGMA reacted with 4,7,10-trioxa-1,13-tridecanediamine to introduce amino-containing spacer arms onto the magnetic support. Glutaraldehyde was then used to activate the amino-functionalized support through aldehyde-mediated coupling, which enabled the subsequent introduction of N,N-bis(carboxymethyl)-L-lysine as an NTA-like chelating ligand. After Ni^2+^ chelation, Ni-NTA groups were formed on the magnetic nanoparticle surface, providing affinity sites for His-tagged AgaDcat immobilization.

### 2.3. Immobilization of β-Agarase AgaDcat

A specified amount of Ni-NTA-MNPs was mixed with AgaDcat solution under shaking to optimize the immobilization conditions. For the immobilization time assay, the mixture was incubated at 30 °C for different durations (0.5 h, 1 h, 2 h, 4 h, and 6 h). For the immobilization temperature assay, the mixture was incubated at different temperatures (20 °C, 25 °C, 30 °C, 35 °C, and 40 °C) for 2 h. The effect of the support-to-enzyme ratio (4:1, 20:1, 40:1, 50:1, 160:1 mg) on immobilization efficiency was also evaluated. The carrier-to-enzyme ratio refers to the mass ratio (*w*/*w*) of Ni-NTA-MNPs to AgaDcat protein, rather than the molecular number ratio. A relatively higher carrier-to-enzyme mass ratio was tested because the effective amount of available Ni-NTA binding sites depends on the surface functionalization density and accessible surface area of the magnetic nanoparticles. After immobilization, the magnetic nanoparticles were separated using an external magnet. The supernatant and washing solutions were collected for residual protein and enzyme activity measurements, while the recovered immobilized enzyme was used for activity determination. The immobilization of AgaDcat was mainly mediated by the coordination interaction between the His-tag of AgaDcat and Ni^2+^ chelated by NTA groups on the magnetic nanoparticle surface. Therefore, the immobilization process was designed as an affinity-mediated strategy rather than nonspecific adsorption or direct glutaraldehyde-mediated covalent crosslinking of the enzyme.

### 2.4. Determination of Free and Immobilized Enzyme Activities

Agarose and 3,5-dinitrosalicylic acid (DNS) used for enzyme activity assays were purchased from Sinopharm Chemical Reagent Co., Ltd. (Shanghai, China). The activity of β-agarase was quantified using the DNS method. Briefly, agarose powder was dispersed in reaction buffer to prepare a freshly mixed 2% (*w*/*v*) agarose suspension. Before the enzymatic assay, the agarose suspension was pre-equilibrated at the corresponding assay temperature and mixed thoroughly to ensure uniform dispersion. For the free enzyme assay, a defined amount of AgaDcat solution was added to the reaction system. For the immobilized enzyme assay, a defined mass of immobilized AgaDcat-Ni-NTA-MNPs was weighed and added to the reaction system. The amount of enzyme used in each reaction was expressed as protein mass. For immobilized AgaDcat, the protein mass was calculated based on the protein loading determined in Section 2.5, while free AgaDcat was added at an equivalent protein mass for comparison. The enzyme was mixed with 2 mL of the freshly prepared agarose suspension, and the reaction mixture was incubated at 30 °C for 5 min. Subsequently, 1.5 mL of DNS reagent was added, and the mixture was boiled for 5 min to terminate the reaction. After cooling on ice, the mixture was diluted to 10 mL with deionized water. A 200 μL aliquot was then taken, and the absorbance at 520 nm was measured to determine the reducing sugar content. One unit of β-agarase activity was defined as the amount of enzyme required to produce 1 μmol of reducing sugar, expressed as D-galactose equivalents, per minute under the assay conditions.

### 2.5. Determination of Protein Loading Efficiency and Activity Retention

The BCA Protein Concentration Determination Kit used for protein quantification was obtained from Beyotime Biotechnology Co., Ltd. (Shanghai, China). The amount of immobilized protein was determined by the indirect subtraction method. Briefly, a known amount of AgaDcat protein was mixed with Ni-NTA-MNPs for immobilization. After immobilization, the magnetic nanoparticles were separated using an external magnet, and the supernatant and washing solutions were collected. The residual protein concentration in the supernatant and washing solutions was determined using the BCA assay. To minimize possible interference from the immobilization support, blank controls containing Ni-NTA-MNPs without enzyme were treated under the same immobilization and washing conditions, and the corresponding absorbance was subtracted during protein quantification.

The amount of immobilized protein was calculated according to the following equation:

Mi = M0 − Ms − Mw

where Mi is the amount of immobilized protein (mg), M0 is the initial amount of AgaDcat added for immobilization (mg), Ms is the amount of residual protein in the supernatant after immobilization (mg), and Mw is the amount of protein detected in the washing solutions (mg). The protein loading efficiency was calculated as follows:

Protein loading efficiency (%) = Mi/M0 × 100

The activity balance during immobilization was also evaluated by measuring the enzyme activity in the initial enzyme solution, the residual supernatant, the washing solutions, and the immobilized enzyme. The immobilized enzyme activity was determined using the standard DNS assay after magnetic recovery and washing of the immobilized enzyme. Blank controls containing Ni-NTA-MNPs without enzyme were included in the activity assay to eliminate the background absorbance of the carrier. The activity retention of the immobilized enzyme was calculated based on the specific activity of the immobilized enzyme relative to that of the same amount of free AgaDcat:

Activity retention (%) = specific activity of immobilized AgaDcat/specific activity of free AgaDcat × 100

The specific activity of free AgaDcat was calculated as the enzyme activity divided by the amount of soluble enzyme protein. For immobilized AgaDcat, the specific activity was calculated based on the amount of immobilized protein on the carrier and expressed as U/mg-protein.

### 2.6. Characterization of Magnetic Nanoparticles and Immobilized Enzyme

Scanning electron microscopy (SEM) and energy-dispersive X-ray spectroscopy (EDS) were performed using a Hitachi Regulus 8100 field-emission scanning electron microscope (Hitachi, Tokyo, Japan) at an accelerating voltage of 5–10 kV. Samples were dispersed on a silicon wafer, coated with platinum under vacuum prior to observation, to characterize the surface morphology and elemental composition and distribution.

Vibrating sample magnetometry (VSM) was carried out with a LakeShore 8604 vibrating sample magnetometer (LakeShore Cryotronics, Westerville, OH, USA) at room temperature (25 ± 2 °C). The magnetic hysteresis loops were recorded from −20,000 to +20,000 Oe to determine the saturation magnetization, coercivity, and superparamagnetic behavior of the samples.

Fourier transform-infrared (FT-IR) spectra of magnetic nanoparticles and immobilized enzymes were obtained by grinding samples with dried potassium bromide, pressing them into thin slices, and scanning with a Thermo Nicolet iS10 FTIR Spectrometer (Thermo Electron Corp., Madison, WI, USA) in the range of 4000 cm^−1^ to 400 cm^−1^. The crystal structure of the magnetic nanoparticles was determined using an X-ray diffractometer (XRD, BRUKER AXS GMBH D8, BRUKER, Karlsruhe, Germany) with a scanning speed of 0.2°/s over a range of 20° to 80°. Thermogravimetric analysis (TGA) was conducted using a METTLER TOLEDO SDTA 851e analyzer (METTLER TOLEDO, Greifensee, Switzerland) from 25 °C to 600 °C at a heating rate of 10 °C/min in a nitrogen atmosphere.

### 2.7. Determination of Enzymatic Properties of Immobilized Enzyme

#### 2.7.1. Optimum Reaction Temperature

For the optimum temperature assay, the reaction was performed in 50 mM Tris-HCl buffer at pH 7.0. The pH of the reaction buffer was measured after equilibration at each assay temperature. A freshly mixed 2% (*w*/*v*) agarose suspension was prepared in the same buffer and pre-equilibrated at each tested temperature before enzyme addition. A defined mass of immobilized AgaDcat-Ni-NTA-MNPs containing a known amount of immobilized protein was added to 2 mL of the agarose suspension and incubated at temperatures ranging from 20 °C to 60 °C for 5 min. Enzyme activity was determined using the DNS method.

#### 2.7.2. Optimal Reaction pH

For the optimum pH assay, freshly mixed 2% (*w*/*v*) agarose suspensions were prepared in buffers with different pH values. The buffer systems were 50 mM sodium acetate buffer for pH 4.0–6.0 and 50 mM Tris-HCl buffer for pH 7.0–9.0. The pH values of the reaction buffers were measured after equilibration at the assay temperature. A defined mass of immobilized AgaDcat-Ni-NTA-MNPs containing a known amount of immobilized protein was added to 2 mL of the agarose suspension and incubated at 40 °C for 5 min. Enzyme activity was determined using the DNS method. Buffer solutions were prepared as follows: 50 mM sodium acetate buffer (pH 4.0–6.0) and 50 mM Tris-HCl buffer (pH 7.0–9.0).

#### 2.7.3. Enzyme Reaction Kinetics

Agarose suspensions at concentrations ranging from 1.0 to 10.0 mg/mL were freshly prepared in reaction buffer and thoroughly mixed before use. The agarose suspensions were mixed with free or immobilized AgaDcat at an equivalent protein mass and reacted for 5 min. The amount of immobilized enzyme was calculated based on the loaded protein content. The Lineweaver–Burk plot was generated by plotting 1/S against 1/V, where S is substrate concentration and V is reaction rate, and *K_m_* and *V_max_* values were calculated. The apparent *k_cat_* value was calculated from *V_max_* using the molecular weight of AgaDcat, which was 40.2 kDa. The apparent catalytic efficiency was expressed as *k_cat_*/*K_m_*. Because agarose is a polysaccharide substrate and its concentration was expressed as mg/mL, *k_cat_*/*K_m_* was reported as mL mg^−1^ s^−1^. Kinetic parameters were obtained from three independent experiments and are presented as mean ± SD. Statistical differences between free and immobilized AgaDcat were analyzed using Student’s t-test, and differences were considered significant at *p* < 0.05.

#### 2.7.4. Reusability

For each reuse cycle, the immobilized enzyme was mixed with 2 mL of freshly prepared 2% (*w*/*v*) agarose suspension and incubated for 5 min under the optimal reaction conditions. After each cycle, the immobilized enzyme was recovered using an external magnetic field, gently washed with reaction buffer to remove residual substrate and products, and then added to a fresh agarose suspension for the next cycle. This process was repeated seven times, and the relative enzyme activity was calculated after each use, with the initial activity defined as 100%.

### 2.8. Statistical Analysis

All enzymatic activity assays, immobilization experiments, and reusability tests were performed in triplicate. Data are presented as the mean ± standard deviation (SD) of three independent experiments. Statistical significance between two groups was analyzed using Student’s *t*-test, while comparisons among multiple groups were performed using one-way analysis of variance (ANOVA) followed by Tukey’s multiple comparison test. Differences were considered statistically significant at *p* < 0.05. Error bars in the figures represent SD.

## 3. Results and Discussion

### 3.1. Immobilization of AgaDcat

**Figure 3:**
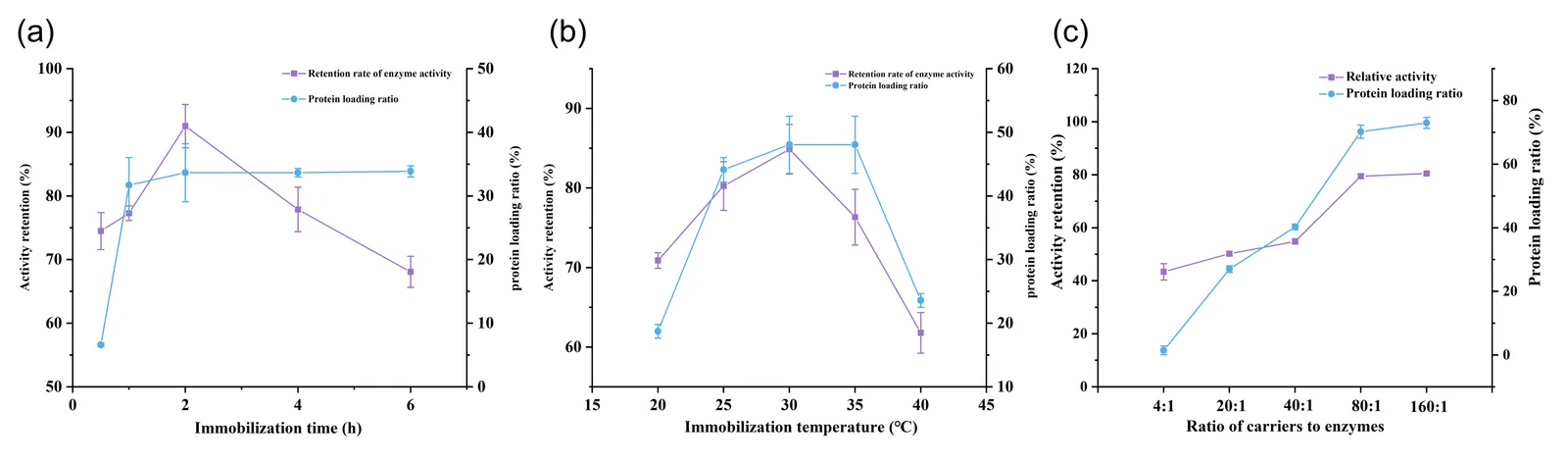
Effects of immobilization time (**a**), immobilization temperature (**b**), and carrier-to-enzyme ratio (**c**) on protein loading efficiency and activity retention. Immobilization was performed under the indicated conditions, and enzyme activity was determined using 2 mL of freshly prepared 2% (*w*/*v*) agarose suspension in 50 mM Tris-HCl buffer (pH 7.0) at 30 °C for 5 min. Activity retention was calculated relative to the specific activity of free AgaDcat, which was defined as 100% (3057 U/mg-protein). Data are presented as mean ± SD (n = 3).

The effects of immobilization time, temperature, and carrier-to-enzyme ratio on the protein loading efficiency and activity retention of the immobilized enzyme are illustrated in Figure 3a–c. During immobilization time optimization, the protein loading efficiency increased rapidly within the first hour and then tended to stabilize, indicating that the available Ni-NTA binding sites on the carrier gradually approached saturation. The highest activity retention was observed after 2 h of immobilization. Further extension of the incubation time did not improve immobilization performance, probably because most accessible binding sites had already been occupied. In addition, prolonged incubation under non-refrigerated shaking conditions may cause partial enzyme inactivation or weak desorption of loosely bound enzyme molecules during subsequent washing, thereby reducing the apparent binding efficiency and activity retention. Therefore, 2 h was selected for subsequent experiments. The immobilization temperature also affected enzyme activity retention, which initially increased and then decreased with increasing temperature, reaching the maximum at 30 °C. In addition, increasing the carrier-to-enzyme ratio improved both protein loading efficiency and activity retention. Here, the carrier-to-enzyme ratio represents the mass ratio of Ni-NTA-MNPs to AgaDcat protein. At low carrier-to-enzyme ratios, the available binding sites may be insufficient, resulting in lower protein loading efficiency and lower activity retention. Increasing the amount of carrier provides more accessible Ni-NTA sites for His-tag binding and improves enzyme immobilization. However, when the carrier-to-enzyme ratio exceeded 80:1, both protein loading efficiency and activity retention tended to plateau, suggesting that the available AgaDcat molecules had been effectively immobilized and that further increasing the carrier amount did not provide additional benefit. After comprehensive optimization, the optimal immobilization condition was determined as a carrier-to-enzyme ratio of 80:1 at 30 °C for 2 h, under which the immobilized enzyme showed a protein loading efficiency of 70.18 ± 1.29% and an activity retention of 80.47%. Compared with previously reported immobilized β-agarase, the activity retention obtained in this study was relatively high. For example, β-agarase immobilized on tannic acid-modified magnetic nanoparticles showed an activity recovery of 39.66%, while agarase immobilized on polydopamine-modified magnetic ferriferous oxide showed an immobilization yield of 63.9% [8,10]. The relatively high activity retention of AgaDcat-Ni-NTA-MNPs may be attributed to the mild His-tag/Ni-NTA affinity interaction, which avoids extensive random modification of the enzyme surface and helps preserve the catalytic conformation of AgaDcat.

### 3.2. Characterization of Magnetic Nanoparticles and Immobilized Enzyme

#### 3.2.1. SEM Morphological Characterization

**Figure 4:**
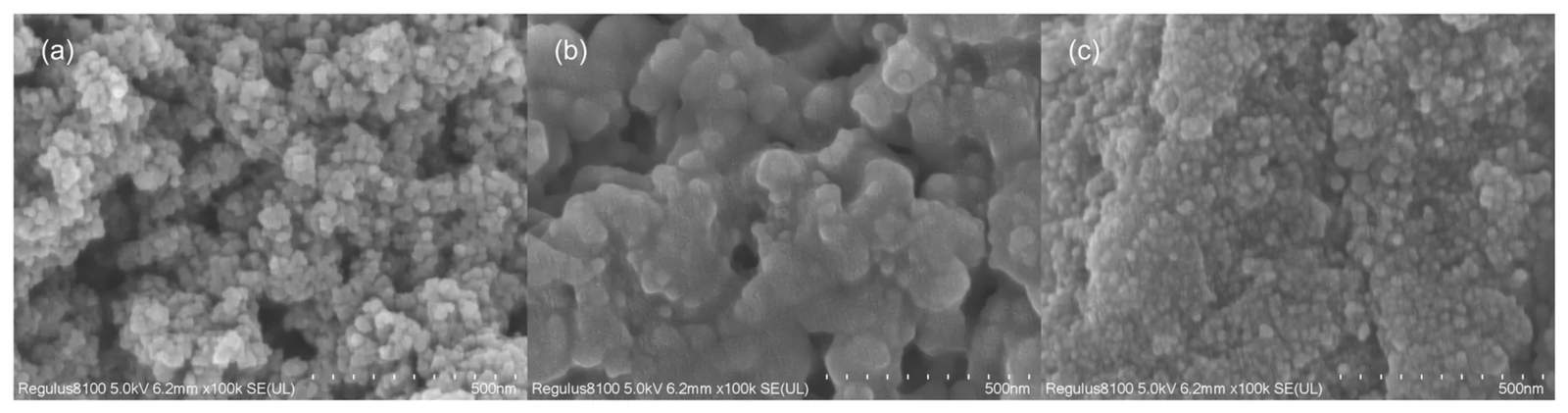
SEM images of Fe_3_O_4_ (**a**), Ni-NTA-MNPs (**b**), and AgaDcat-Ni-NTA-MNPs (**c**).

The morphological changes in magnetic nanoparticles during preparation and enzyme immobilization were observed by SEM at a scale of 500 nm, as shown in Figure 4. The bare Fe_3_O_4_ nanoparticles presented a regular spherical morphology with slight agglomeration, which is consistent with the typical morphology of Fe_3_O_4_ prepared by the co-precipitation method. After multi-step modification, Ni-NTA-MNPs showed improved dispersion and a smoother and denser surface, indicating the successful grafting of organic layers and NTA chelating structures onto the Fe_3_O_4_ core. After AgaDcat immobilization, the surface of AgaDcat-Ni-NTA-MNPs became rougher, with visible protrusions and coating layers. Slight adhesion between particles was also observed, which may be attributed to intermolecular interactions among immobilized protein molecules. These morphological changes provide visual evidence for the successful immobilization of His-tagged AgaDcat onto Ni-NTA-MNPs.

#### 3.2.2. EDS Elemental Composition and Distribution Analysis

**Figure 5:**
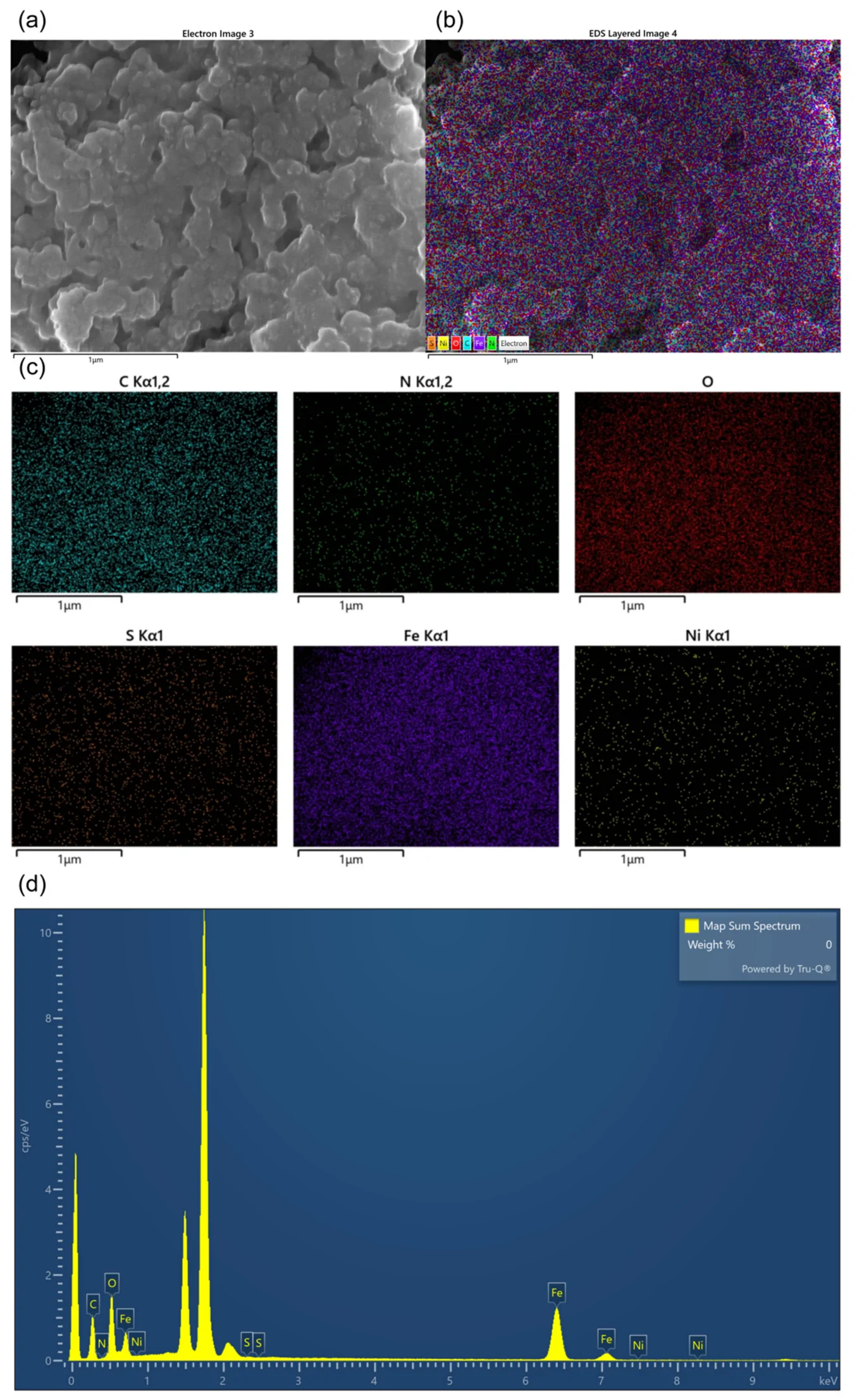
EDS mapping analysis of Ni-NTA-MNPs. (**a**) SEM image; (**b**) merged elemental mapping image; (**c**) individual elemental maps of C, N, O, S, Fe, and Ni; and (**d**) EDS spectrum.

Energy-dispersive X-ray spectroscopy (EDS) and elemental mapping were conducted to verify the elemental composition and distribution of Ni-NTA-MNPs, as shown in Figure 5. The EDS mapping results (Figure 5b,c) clearly demonstrate the presence and homogeneous dispersion of Fe, C, N, O, S, and Ni elements across the entire Ni-NTA-MNPs matrix. Fe is the most abundant element, originating from the Fe_3_O_4_ magnetic core and serving as the structural foundation of the carrier, whose uniform distribution confirms the integrity of the magnetic core during multi-step modification. C and O are the main components of the organic shell, derived from the poly (glycidyl methacrylate) (PGMA) polymer layer, oleic acid modifier, and nitrilotriacetic acid (NTA) chelating ligand, verifying the successful grafting of the organic functional layer onto the Fe_3_O_4_ core. N is mainly introduced by 4,7,10-trioxo-1,13-tridecanediamine (TDA) and N,N-bis(carboxymethyl)-L-lysine, where TDA provides amino groups for glutaraldehyde crosslinking, and the latter is the key precursor for constructing the NTA chelating structure. The trace S signal can be attributed to residual ammonium persulfate used as an initiator in the PGMA polymerization step. Most importantly, the distinct and uniformly distributed Ni signal directly confirmed that Ni^2+^ ions were successfully chelated by the NTA groups on the particle surface. The EDS spectrum further verified the elemental composition of Ni-NTA-MNPs, with Fe accounting for the highest mass fraction, consistent with the magnetic core structure. The detection of Ni and N provided chemical evidence for the successful construction of the Ni-NTA chelating layer, laying a foundation for the subsequent specific immobilization of His-tagged AgaDcat.

#### 3.2.3. VSM Magnetic Property Analysis

**Figure 6:**
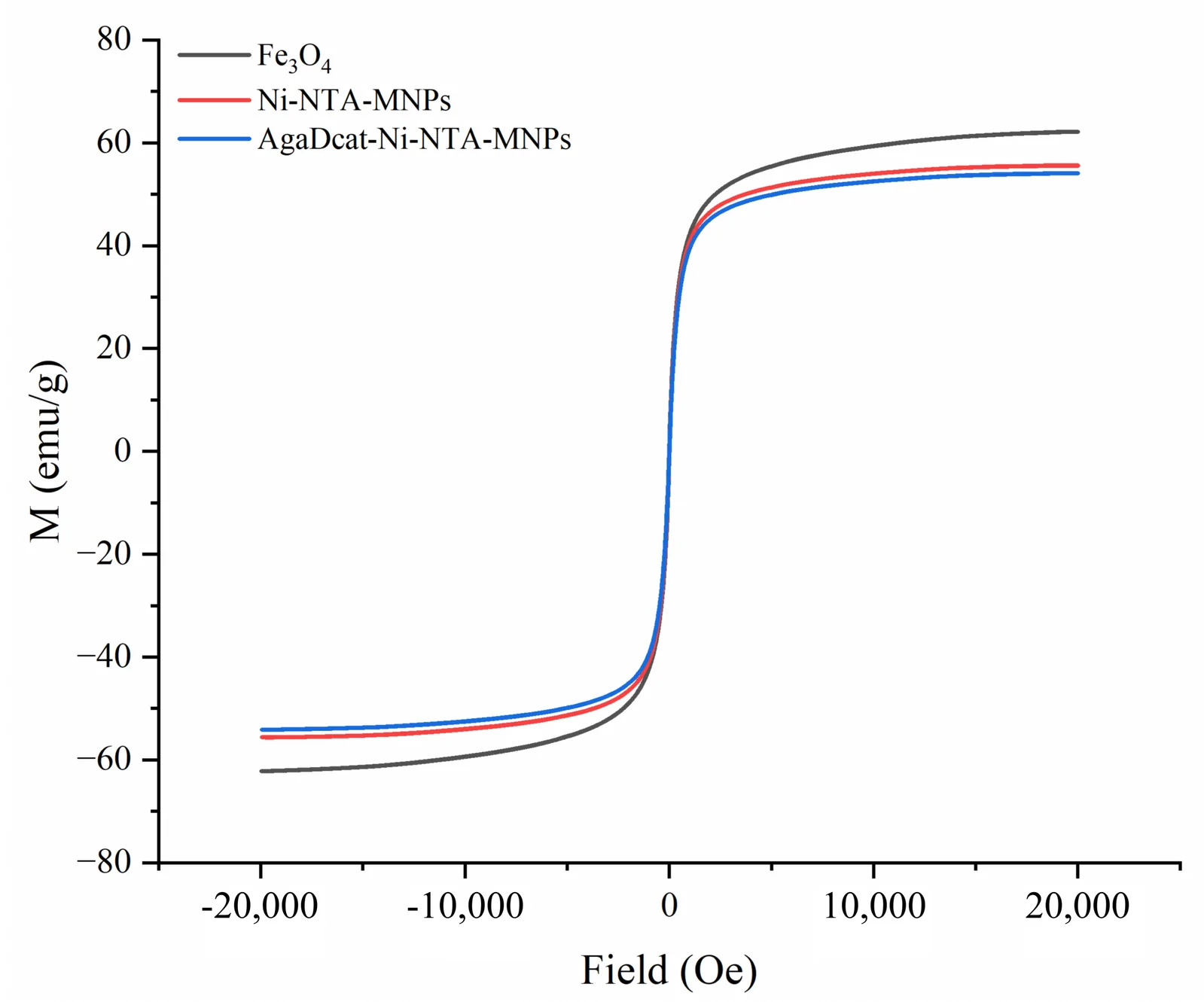
Magnetic hysteresis loops of Fe_3_O_4_, Ni-NTA-MNPs, and AgaDcat-Ni-NTA-MNPs measured by VSM.

Vibrating sample magnetometry (VSM) was performed to evaluate the magnetic responsiveness of Fe_3_O_4_, Ni-NTA-MNPs, and AgaDcat-Ni-NTA-MNPs. The corresponding hysteresis loops are presented in Figure 6. All three samples exhibited typical S-shaped magnetization curves with negligible coercivity and remanence, indicating their superparamagnetic behavior throughout the multi-step modification and enzyme immobilization processes [24].

As shown in Figure 6, the bare Fe_3_O_4_ nanoparticles displayed the highest saturation magnetization (Ms) value, reaching approximately 62 emu/g. After the grafting of organic layers and NTA chelating groups onto magnetic nanoparticles to form Ni-NTA-MNPs, the Ms value slightly decreased to around 56 emu/g. This reduction in magnetic responsiveness can be attributed to the non-magnetic organic shell formed on the Fe_3_O_4_ surface, which increases the thickness of the non-magnetic layer and reduces the effective magnetic volume per unit mass. Following the immobilization of AgaDcat, the Ms value of AgaDcat-Ni-NTA-MNPs further decreased slightly to approximately 55 emu/g. This minor decline is likely due to the protein layer covering the particle surface, which adds another non-magnetic coating and increases the overall size of the nanoparticles.

Although the Ms values slightly decreased after surface modification and enzyme immobilization, both Ni-NTA-MNPs and AgaDcat-Ni-NTA-MNPs maintained sufficient magnetic responsiveness. This superparamagnetic property enables the nanoparticles to be rapidly separated from the reaction mixture under an external magnetic field, while redispersion easily occurs when the magnetic field is removed. These characteristics confirm that Ni-NTA-MNPs are suitable for practical enzyme immobilization and industrial biocatalytic applications, providing convenient and efficient separation of the biocatalyst.

#### 3.2.4. FT-IR, TGA, and XRD Characterization

**Figure 7:**
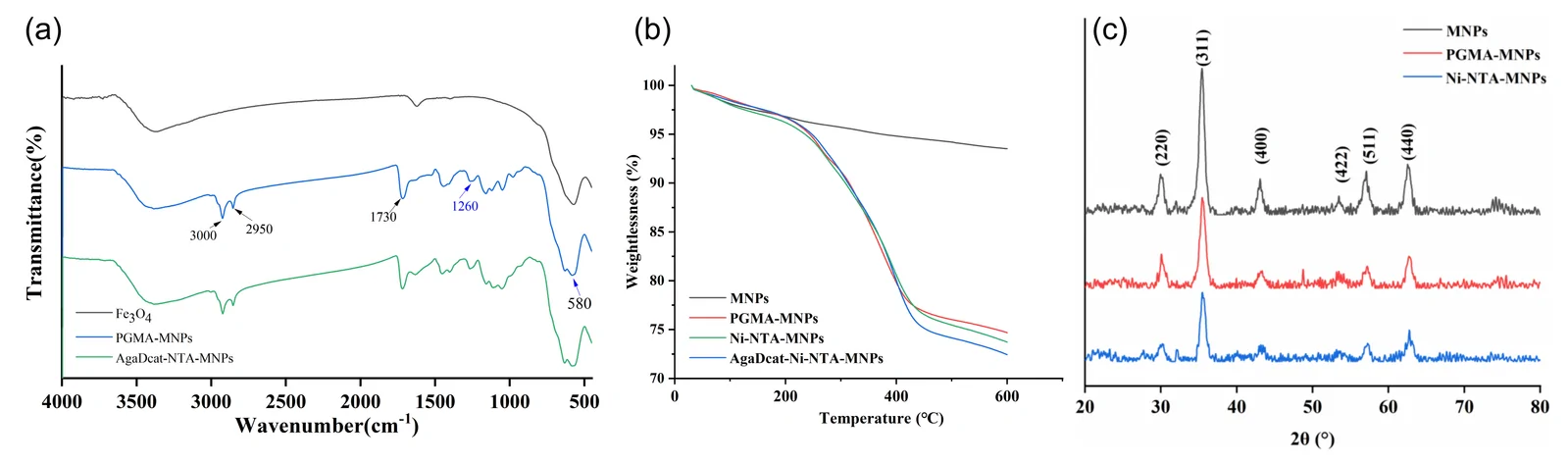
FT-IR spectra (**a**), TGA curves (**b**), and XRD patterns (**c**) of Fe_3_O4 nanoparticles, PGMA-MNPs, Ni-NTA-MNPs, and AgaDcat-Ni-NTA-MNPs.

To confirm the successful preparation of the immobilized enzyme, FT-IR, TGA, and XRD characterizations were conducted. As shown in Figure 7a, the FT-IR spectra of all magnetic nanoparticles exhibited a characteristic absorption peak near 580 cm^−1^, corresponding to Fe–O bond vibrations and confirming the presence of Fe_3_O_4_. The strong peak at 1730 cm^−1^ in PGMA-MNPs was assigned to the carbonyl group of the PGMA moiety, while the peaks at 2950 and 3000 cm^−1^ were attributed to methylene groups [25,26]. The absorption peak at 1260 cm^−1^ was characteristic of the epoxide ring [27]. After immobilization, the characteristic absorption band at 1600–1700 cm^−1^, corresponding to amide I vibrations, appeared in AgaDcat-Ni-NTA-MNPs, indicating the successful immobilization of β-agarase AgaDcat onto the magnetic nanoparticles [23].

As shown in Figure 7b, the thermal weight loss curves further confirmed the stepwise surface modification and enzyme immobilization. The initial weight loss below 200 °C was mainly attributed to the evaporation of adsorbed water on the particle surfaces. From 20 °C to 600 °C, the weight losses of PGMA-MNPs, Ni-NTA-MNPs, and AgaDcat-Ni-NTA-MNPs were higher than that of bare Fe_3_O_4_ nanoparticles, mainly due to the decomposition of the polymer shell, chelating ligands, and immobilized protein layer [28]. The increased weight loss after AgaDcat immobilization further supported the successful loading of the enzyme onto Ni-NTA-MNPs.

As shown in Figure 7c, the characteristic diffraction peaks of Fe_3_O_4_ were observed at 2θ = 30.12°, 35.50°, 43.12°, 53.50°, 57.03°, and 62.63°, corresponding to the (220), (311), (400), (422), (511), and (440) crystal planes of magnetite Fe_3_O_4_, respectively. These diffraction peaks were consistent with the standard Fe_3_O_4_ crystal structure [9], indicating that the magnetic core maintained its crystalline structure during surface modification and enzyme immobilization.

Overall, the changes observed in SEM, EDS, VSM, FT-IR, TGA, and XRD analyses were consistent with previous reports on magnetic nanoparticle-based enzyme immobilization systems. The appearance of rougher particle surfaces after enzyme immobilization, the detection of Ni and N signals, the decrease in saturation magnetization after organic modification and protein loading, and the additional thermal weight loss caused by the organic and protein layers have all been regarded as typical evidence for successful surface functionalization and enzyme immobilization [8,9,10,23,24,28]. These results collectively confirmed the successful preparation of Ni-NTA-MNPs and the subsequent immobilization of AgaDcat.

The chemical mechanism for support functionalization and enzyme immobilization is further illustrated in Figure 2. During support preparation, PGMA provided reactive epoxy groups on the Fe_3_O_4_ magnetic nanoparticle surface. These epoxy groups reacted with amino-containing spacer arms, thereby introducing surface amino groups for further functionalization. Glutaraldehyde was then used as a bifunctional linker to activate the amino-functionalized support, allowing the introduction of N,N-bis(carboxymethyl)-L-lysine and the formation of NTA-like chelating groups. Subsequent Ni^2+^ chelation generated Ni-NTA affinity sites on the magnetic nanoparticle surface. The immobilization of AgaDcat was mainly achieved through coordination between the His-tag of AgaDcat and Ni^2+^ chelated by NTA groups, providing a relatively selective and mild affinity-based immobilization route.

Although conventional glutaraldehyde-mediated enzyme immobilization often involves the ε-amino groups of surface Lys residues, this mechanism is not applicable to the present immobilization step. In this study, glutaraldehyde was used during support functionalization to activate the amino-functionalized magnetic nanoparticles and enable the subsequent introduction of N,N-bis(carboxymethyl)-L-lysine as an NTA-like chelating ligand. During enzyme immobilization, AgaDcat was mixed with the preformed Ni-NTA-MNPs without additional glutaraldehyde. Therefore, Lys-mediated covalent crosslinking of AgaDcat was not expected to be the immobilization mechanism. Instead, AgaDcat immobilization was mainly attributed to the coordination interaction between the His-tag of AgaDcat and Ni^2+^ chelated by NTA groups on the magnetic nanoparticle surface.

### 3.3. Enzymatic Properties of Immobilized β-Agarase AgaDcat

**Figure 8:**
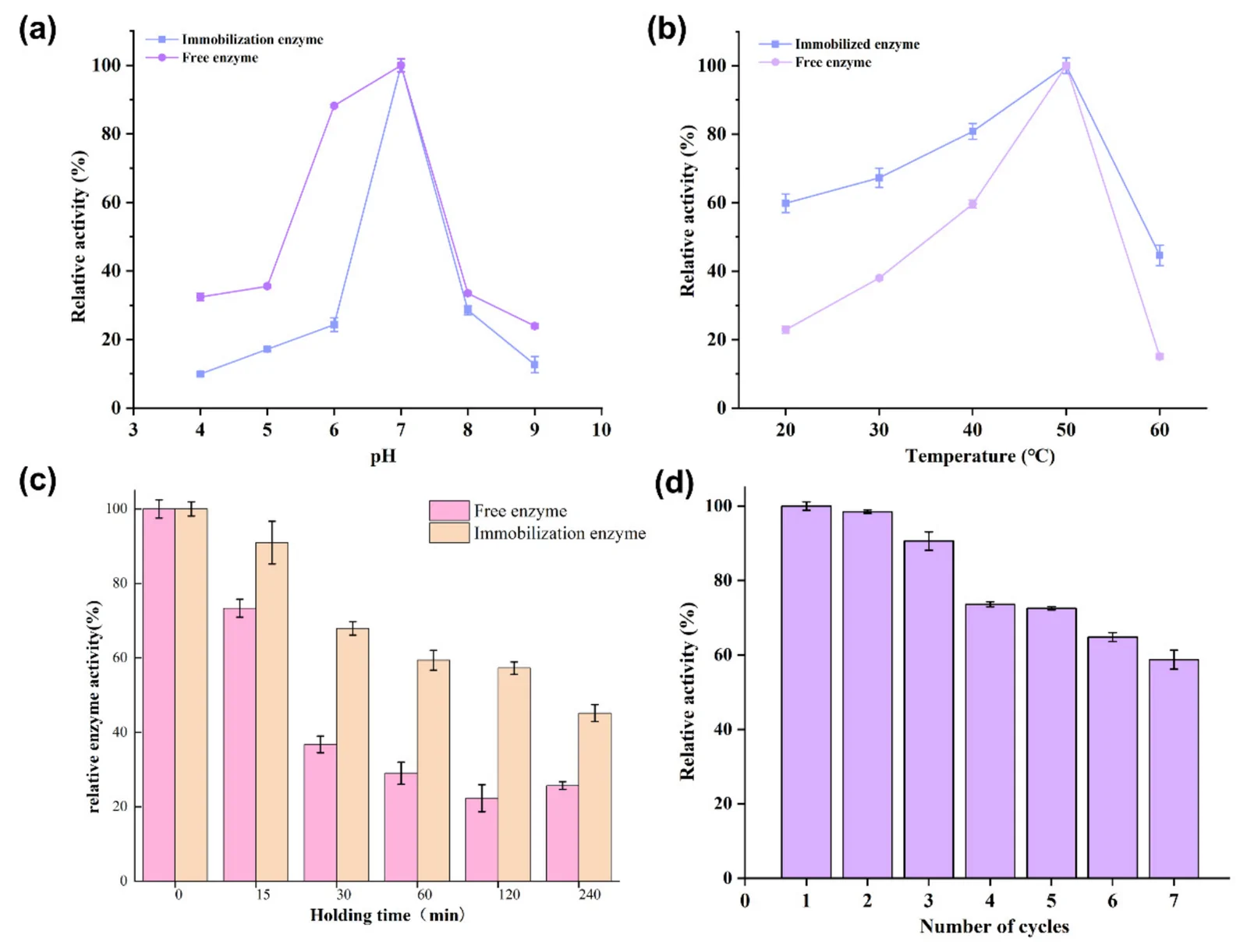
Effects of pH (**a**) and temperature (**b**) on the activities of free and immobilized AgaDcat; thermal stability at 50 °C (**c**); and reusability of immobilized AgaDcat (**d**). Enzyme activity was measured using 2 mL of freshly prepared 2% (*w*/*v*) agarose suspension for 5 min under the corresponding assay conditions. The pH assay was performed at pH 4.0–9.0 and 40 °C, while the temperature assay was performed at 20–60 °C in 50 mM Tris-HCl buffer at pH 7.0. For thermal stability and reusability assays, residual activity was calculated by defining the initial activity before heat treatment or the activity in the first cycle as 100%, respectively. Under the standard assay conditions, the specific activities of free and immobilized AgaDcat were 3057 and 2460 U/mg-protein, respectively. Data are presented as mean ± SD (n = 3).

The effects of pH and temperature on the activities of free and immobilized AgaDcat are shown in Figure 8a and Figure 8b, respectively. Relative activity in the pH and temperature assays was normalized to the activity measured under the corresponding optimal condition, whereas residual activity in the thermal stability and reusability assays was normalized to the initial activity before heat treatment and the activity in the first cycle, respectively. Under the standard assay conditions, the specific activities of free and immobilized AgaDcat were 3057 and 2460 U/mg-protein, respectively. Both free and immobilized enzymes exhibited the highest activity at pH 7.0. Under acidic conditions, the free enzyme showed higher residual activity than the immobilized enzyme, whereas both enzymes displayed comparable tolerance under alkaline conditions. As shown in Figure 8b, the optimal reaction temperature for both free and immobilized AgaDcat was 50 °C. Notably, the immobilized enzyme exhibited better temperature tolerance than the free enzyme, especially at lower temperatures of 20–40 °C and at the higher temperature of 60 °C.

The thermal stability of free and immobilized enzymes is depicted in Figure 8c. For the thermal inactivation assay, the initial specific activity before heat treatment was defined as 100%. At 50 °C, the immobilized AgaDcat demonstrates significantly enhanced thermal stability compared to the free enzyme. After 60 min of incubation at 50 °C, the relative activity of the free enzyme drops to 29.1 ± 0.7%, whereas the immobilized AgaDcat retains 59.4% of its initial activity. Furthermore, after 240 min at 50 °C, the free and immobilized enzymes retain 25.7% and 45.2% of their relative activities, respectively. This enhanced residual activity after immobilization is likely related to the stabilizing effect of enzyme immobilization on the protein structure [29]. Immobilization may help maintain the active conformation of AgaDcat during thermal incubation. It may also reduce irreversible aggregation or thermal unfolding of the enzyme, thereby improving its tolerance to heat treatment. Therefore, the higher activity observed in Figure 8c should be interpreted mainly as improved residual activity and thermal stability after immobilization, rather than a simple increase in the intrinsic catalytic activity of the enzyme. The specific activity and apparent kinetic parameters of free and immobilized AgaDcat are shown in Table 1. The specific activities of free and immobilized AgaDcat were 3057 and 2460 U/mg-protein, respectively. Compared with the free enzyme, immobilized AgaDcat showed a lower *K_m_* value, decreasing from 6.36 to 2.65 mg/mL, suggesting an improved apparent affinity toward agarose after immobilization. This decrease in *K_m_* may be related to the local enrichment of agarose around the immobilized enzyme or a more favorable enzyme orientation mediated by the His-tag/Ni-NTA interaction. However, the *V_max_* value decreased from 3274.96 to 2021.92 U/mg. This reduction may be attributed to mass-transfer limitations, steric hindrance between the immobilized enzyme and the macromolecular agarose substrate, or restricted conformational flexibility after immobilization. Therefore, the kinetic parameters of immobilized AgaDcat should be interpreted as apparent values affected by both enzyme–substrate interaction and heterogeneous diffusion effects. Similar kinetic behaviors, including altered *K_m_* and reduced maximum reaction rate after immobilization, have also been reported in other immobilized agarase systems based on tannic acid-modified Fe_3_O_4_ nanoparticles and polydopamine-modified magnetic carriers [8,10], as well as in enzymes immobilized on metal-chelated magnetic carriers [30].

The excellent reusability of immobilized enzymes is advantageous for industrial applications. As shown in Figure 8d, the immobilized enzyme retains 90.59% of its initial activity after three cycles and maintains 58.71% after seven cycles. In the reusability assay, the specific activity of immobilized AgaDcat in the first cycle was defined as 100%, corresponding to 2460 U/mg-protein. The decline in activity after repeated use may be attributed to changes in enzyme conformation or enzyme leakage [31]. Compared to other methods, such as dopamine polymerization-modified MOF immobilized agarase and tannic acid-modified MNPs immobilized β-agarase, which retain less than 50% and 22.5% of their initial activities after seven uses respectively [8,10], the IMAC-based immobilization of AgaDcat demonstrates superior reusability. The improved reusability of immobilized AgaDcat is particularly important for the repeated-batch production of functional NAOS, because enzyme cost is one of the key factors limiting the practical application of soluble agarases. Compared with previously reported immobilized agarase systems, including dopamine-polymerized MOF-based carriers and tannic acid-modified magnetic nanoparticles, the present IMAC-based immobilization strategy showed better activity retention after repeated use [8,10]. In addition, magnetic separation allows rapid recovery of the biocatalyst from the reaction mixture, which may simplify downstream processing and reduce enzyme loss during NAOS preparation [31]. These advantages indicate that AgaDcat-Ni-NTA-MNPs have potential for efficient and reusable production of neoagarooligosaccharides. To further evaluate the performance of the present immobilized enzyme system, a detailed comparison with previously reported immobilized β-agarase systems is provided in Table 2 [8,9,10,29,32,33,34]. Because different studies used different enzyme sources, support materials, immobilization chemistries, substrate concentrations, activity definitions, and reaction conditions, direct comparison among immobilized β-agarase systems should be interpreted carefully [8,9,10,29,32,33,34]. Therefore, the comparison focused on final performance indicators, including activity retention after immobilization, residual activity after repeated use, operational stability, and product formation, rather than on a single activity parameter. Under the optimized conditions, AgaDcat-Ni-NTA-MNPs retained 80.47% of the free enzyme activity after immobilization and maintained 58.71% of its initial activity after seven reuse cycles. In addition, the immobilized enzyme reached reaction equilibrium within 4–8 h and mainly produced NA4 and NA6, further supporting its potential for repeated-batch neoagarooligosaccharide production. The lower apparent *K_m_* value of immobilized AgaDcat may be associated with local substrate enrichment near the carrier surface, whereas the reduced *V_max_* may result from mass-transfer limitation and restricted conformational flexibility, which are common phenomena in immobilized enzyme systems [29,30].

Different studies used different enzyme sources, substrates, activity definitions, and assay conditions; therefore, the comparison mainly focuses on immobilization strategy, catalytic performance, stability, reusability, and application performance.

### 3.4. Degradation Products Analysis

**Figure 9:**
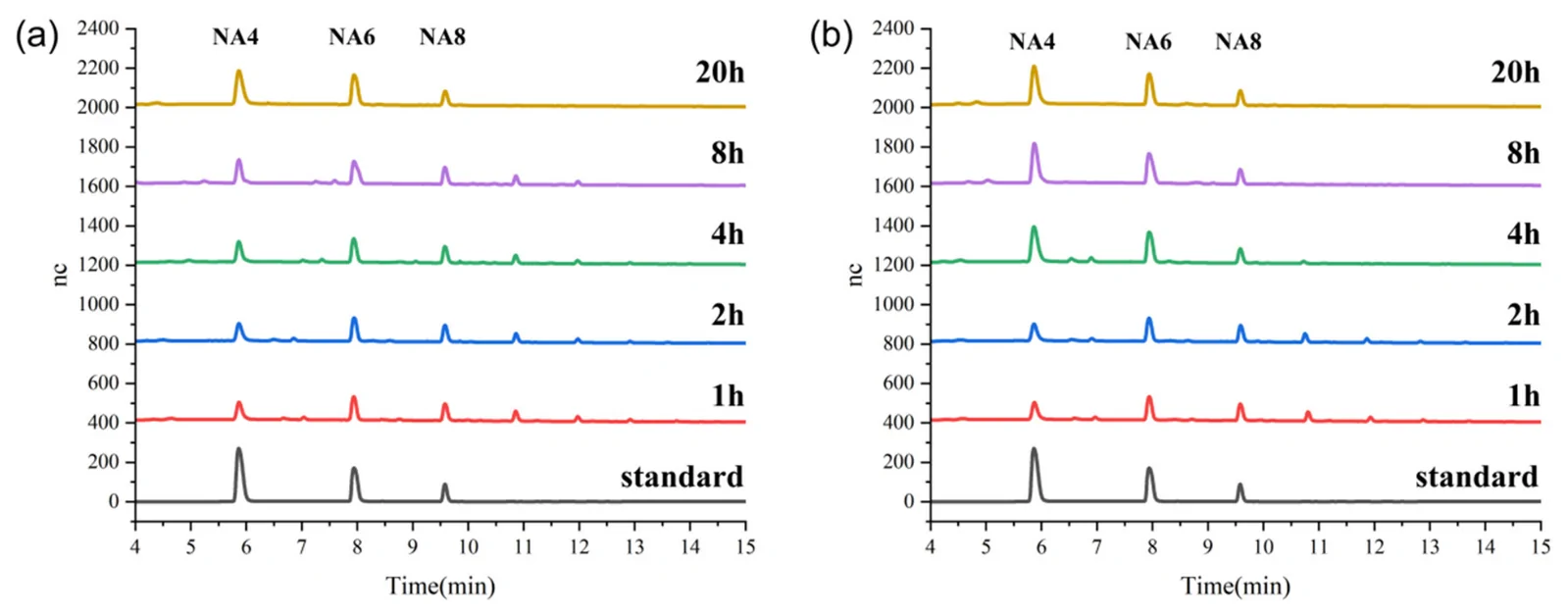
HPAEC-PAD chromatograms of hydrolysis products generated by free AgaDcat (**a**) and AgaDcat-Ni-NTA-MNPs (**b**) at different reaction times. Hydrolysis reactions were performed using 2% (*w*/*v*) agarose suspension as the substrate at pH 7.0 and 50 °C, with free and immobilized AgaDcat added at an equivalent protein mass. The hydrolysis products were analyzed by HPAEC-PAD to determine the distribution of NA4, NA6, and NA8.

The hydrolysis products of free and immobilized AgaDcat at different reaction times were analyzed by high-performance anion-exchange chromatography with pulsed amperometric detection (HPAEC-PAD), as shown in Figure 9. The major products for both free and immobilized enzymes were NA4, NA6, and NA8, in accordance with the endo-type cleavage pattern of AgaDcat that hydrolyzes β-1,4-glycosidic linkages in agarose to generate neoagarooligosaccharides (NAOS) with distinct degrees of polymerization.

As displayed in Figure 9a, the free enzyme showed a relatively slow hydrolysis rate. Trace high-molecular-weight agarose residues and a high level of the intermediate NA8 were still detectable even after 8 h, indicating that the reaction had not reached equilibrium. The chromatographic profile suggested that the reaction approached completion at 20 h, with a stable product composition dominated by NA4 and NA6.

In contrast, immobilized AgaDcat exhibited significantly improved catalytic efficiency and reaction rate (Figure 9b). No residual high-molecular-weight agarose was detected after an 8 h reaction. Given that the NA8 content had already declined to a low level by 4 h and the product profile remained consistent with that at 20 h, it can be concluded that the reaction reached equilibrium within 4–8 h. At equilibrium, NA4 and NA6 were the primary end products, matching the product distribution of the free enzyme at 20 h. The reduction in reaction time is attributed to immobilization, which enhances the thermostability of AgaDcat and mitigates its irreversible inactivation under reaction conditions, thereby augmenting overall catalytic performance.

These results demonstrate that immobilization of AgaDcat onto Ni-NTA-MNPs via IMAC effectively enhances its catalytic performance, enabling rapid and efficient hydrolysis of agarose into NAOS, which is highly favorable for the industrial production of neoagarooligosaccharides. From the perspective of food-related applications, the proposed immobilized biocatalytic system provides several potential advantages for the preparation of functional neoagarooligosaccharides. First, the magnetic Ni-NTA-MNPs can be rapidly separated from the hydrolysate using an external magnetic field, which may help reduce residual enzyme and carrier particles in the product stream. Second, immobilization enables repeated use of AgaDcat, thereby decreasing enzyme consumption and improving process economy. Third, the shortened reaction time may reduce prolonged thermal exposure and improve the operational efficiency of NAOS preparation. Nevertheless, several limitations should be further considered before direct food-grade application. First, because Ni^2+^ is used as the chelated metal ion in the Ni-NTA system, potential Ni^2+^ leaching during repeated operation should be quantitatively evaluated, especially under long-term hydrolysis conditions. Second, although magnetic separation can facilitate catalyst recovery, residual magnetic particles in the hydrolysate should be carefully monitored and removed to meet food-processing safety requirements. Third, the present study evaluated the immobilized enzyme mainly in repeated-batch reactions, whereas long-term operational stability under continuous or pilot-scale conditions remains to be verified. In addition, large-scale application would require further optimization of nanoparticle preparation, enzyme immobilization cost, carrier regeneration, and separation efficiency. Therefore, although the present system demonstrates promising process feasibility, additional leaching tests, long-term operation studies, scale-up validation, and safety assessment are still necessary before its application in food manufacturing.

## 4. Conclusions

A key challenge in the enzymatic production of neoagarooligosaccharides is the lack of a reusable and easily recoverable β-agarase biocatalyst that can maintain catalytic performance while reducing enzyme loss during repeated operation. This study addressed this gap by developing an IMAC-based magnetic immobilization strategy for His-tagged β-agarase AgaDcat using Ni-NTA-functionalized magnetic nanoparticles. Unlike nonspecific adsorption or random covalent immobilization, the His-tag/Ni-NTA affinity interaction provides a mild and more selective immobilization route, which helps preserve enzyme activity while enabling rapid magnetic recovery.

The Ni-NTA-MNPs can serve as an effective affinity-based support for preparing a recyclable β-agarase biocatalyst for functional neoagarooligosaccharide production. The immobilized AgaDcat showed improved operational stability, reusability, and apparent substrate affinity and accelerated the production of NA4 and NA6 as the major products. These findings support the feasibility of affinity-mediated magnetic immobilization as a practical strategy for improving β-agarase utilization in repeated-batch agarose hydrolysis.

Nevertheless, several scientific and practical questions remain unresolved. First, after Ni^2+^ chelation, thorough washing is required to remove unbound or weakly adsorbed Ni^2+^ ions and to minimize free Ni^2+^ residues on the immobilization support, particularly for food-related applications. Second, the long-term stability of the immobilized enzyme under continuous or pilot-scale hydrolysis conditions remains to be verified. Third, carrier regeneration, immobilization cost, magnetic separation efficiency, and possible residual magnetic particles in the product stream require further assessment.

## Figures and Tables

**Table 1:** Specific activity and apparent kinetic parameters of free and immobilized AgaDcat.

Sample	Specific Activity (U/mg-Protein)	*K_m_* (mg/mL)	*V_max_* (U/mg)	*k_cat_* (s^−1^)	*k_cat_*/*K_m_* (mL mg^−1^ s^−1^)
AgaDcat	3057 ± 1.21 ^a^	6.36 ± 0.72 ^a^	3274.96 ± 0.12 ^a^	2194.22 ± 0.08 ^a^	345.00 ± 39.06 ^a^
Immobilized AgaDcat	2460 ± 0.87 ^b^	2.65 ± 0.31 ^b^	2021.92 ± 0.05 ^b^	1354.69 ± 0.03 ^b^	511.20 ± 59.80 ^b^

Data are presented as mean ± SD (n = 3). Different superscript lowercase letters within the same column indicate significant differences between free and immobilized AgaDcat (*p* < 0.05).

**Table 2:** Comparison of the present immobilized AgaDcat system with previously reported immobilized β-agarase systems.

Enzyme/Support System	Immobilization Strategy	Optimum pH/Temperature	Activity or Kinetic Performance	Stability/Reusability	Main Products or Application	Reference
AgaDcat-Ni-NTA-MNPs	IMAC-based His-tag/Ni-NTA affinity immobilization on magnetic nanoparticles	pH 7.0/50 °C	Specific activity: 2460 U/mg-protein; *K_m_*: 2.65 mg/mL; *V_max_*: 2021.92 U/mg; activity retention: 80.47%	90.59% activity after 3 cycles; 58.71% after 7 cycles	NA4 and NA6 as major products; reaction equilibrium within 4–8 h	This study
β-Agarase-TA-MNPs	Tannic acid-modified Fe_3_O_4_ magnetic nanoparticles	pH 7.5/50 °C	Activity recovery: 39.66% under optimized enzyme loading; *K_m_*: 1.54 mg/mL; *V_max_*: 0.044 mg/(min·mL)	77% residual activity after 1 h at 60 °C; 22.5% activity after 7 cycles	Preparation of neoagaro-oligosaccharides with different average polymerization degrees and antioxidant activities	Xiao et al. [8]
β-Agarase-BTPEG24-SA@MNPs	Biotin/streptavidin-mediated immobilization on streptavidin-coated Fe_3_O_4_ nanoparticles with PEG24 linker	pH 7.0/30 °C	Specific activity: 16.18 U/mg; activity retention: 135.67%; *k_cat_*: 39.80 s^−1^	*t_1/2_*: 1155.25 min at 40 °C, 231.05 min at 45 °C, and 154.03 min at 50 °C; >50% activity after 7 cycles	Neoagarobiose production; final yield 12,719.15 μM, 1.89-fold higher than free enzyme	Liu et al. [9]
Agarase-MFO-PDA	Polydopamine-assisted immobilization on magnetic ferriferous oxide	Kinetic assay at pH 7.5 and 30 °C	Average immobilization yield: 63.9%; *K_m_*: 4.73 mg/mL; *V_m_*: 175.13 U/(mL·min)	>50% activity after 5 cycles; >27% activity after 9 cycles	Reusable agarase system for agar degradation, mainly producing neoagarotetraose	Zhang et al. [10]
Amberlite IRA-900 immobilized agarase	Ionic bonding/crosslinking immobilization on glutaraldehyde-activated Amberlite IRA-900	pH 8.0/45 °C	Activity maintained under alkaline conditions; immobilized enzyme showed higher thermal tolerance than free enzyme	90% activity after 8 cycles; approximately 50% activity after 12 cycles; stored for 3 months as wet beads and >6 months as dry beads	Repeated-batch production of neoagarobiose, neoagarotetraose, and neoagarohexaose	Koti et al. [29]
Aga2Y5N-TP@Lys@Fe_3_O_4_	Tyrosinase-catalyzed phenol-mediated immobilization on L-lysine-coated magnetic particles	Broad activity range: pH 4–9 and 25–75 °C	Recovered activity: 80%; *V_max_* and *k_cat_* were 73% of free Aga2Y5N	~80% activity after 180 min at 65 °C; 92% activity after 86 days at 4 °C; 65% activity after 11 cycles	NA4 and NA6 production from *Gelidium amansii*; maximum NAOS production of 335 μmol/g after 48 h at 45 °C	Weldemhret et al. [32]
Coimmobilized AgWH50B/K134D-CC-Fe_3_O_4_@SiO_2_	Covalent coimmobilization of β-agarase and α-neoagarobiose hydrolase on trichlorotriazine-functionalized silica-coated magnetic nanoparticles	pH 7.0 for immobilized AgWH50B; optimal coimmobilized reaction at 27.5 °C	One-pot cascade conversion; coimmobilized system showed higher agarose-to-L-AHG conversion efficiency than free agarases	46.4% activity retained after 8 cycles	L-AHG and AO3 production; L-AHG yield: 40.6%, 6.5% higher than free agarases	Wang et al. [33]
Site-specifically immobilized Aga50D-MAL-MNPs	Site-specific covalent immobilization of engineered Cys mutants on maleimide-modified magnetic nanoparticles	Optimal reaction conditions not changed compared with free enzyme	Activity retention: 96.7% for R66C-MAL-MNPs and 96.9% for K588C-MAL-MNPs; protein immobilization rate >40%	*t*_1/2_ increased up to 21.25-fold at 40 °C; all immobilized enzymes retained >90% activity in 50% acetone	Precise immobilization strategy for improving β-agarase thermal and solvent stability	Liu et al. [34]

## Data Availability

The original contributions presented in this study are included in the article/Supplementary Materials. Further inquiries can be directed to the corresponding author.

## Supplementary Materials

The following supporting information can be downloaded at: https://www.mdpi.com/article/10.3390/foods15162778/s1, Figure S1. Plasmid map of pET-28a(+)-AgaDcat used for recombinant expression of His-tagged β-agarase AgaDcat.
