# Supplementary material for: Immobilization of β-Agarase onto Ni-NTA Magnetic Nanoparticles for Efficient Production of Functional Neoagarooligosaccharides
Source: Foods. 2026 Aug 7;15(16):2778. doi: 10.3390/foods15162778 (PMC13512263; doi:10.3390/foods15162778)
Supplement: Supplementary file 1 [file foods-15-02778-s001.zip › foods-4489197-supplementary.pdf]

Supplementary

Immobilization of β-Agarase onto Ni-NTA Magnetic Nanoparticles for Efficient Production of Functional Neoagarooligosaccharides

Kaifan Qiu [1,2,3], Chen Wang [1,2,3], Xingfei Li [1,2,3], Zhengyu Jin [1,2,3] and Jie Long [1,2,3,*]

[1] The State Key Laboratory of Food Science and Technology, Jiangnan University, 1800 Lihu Road, Wuxi 214122, China
[2] School of Food Science and Technology, Jiangnan University, 1800 Lihu Road, Wuxi 214122, China
[3] Collaborative Innovation Center of Food Safety and Quality Control in Jiangsu Province, Jiangnan University, Wuxi 214122, China
* Correspondence: jielong@jiangnan.edu.cn; Tel./Fax: +86-510-85320225:

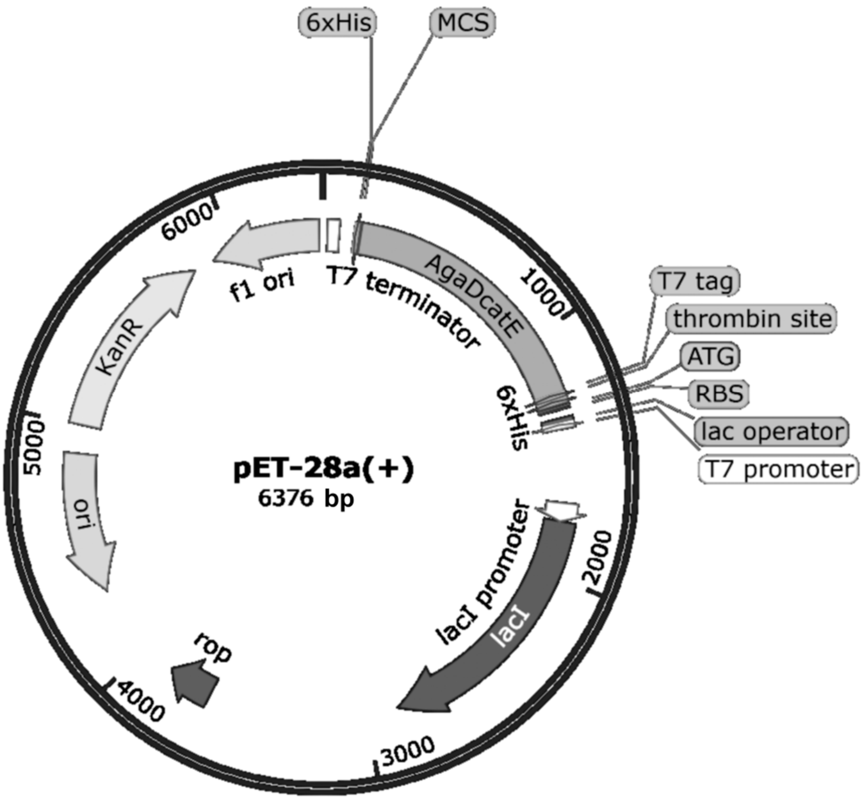

Figure S1. Plasmid map of pET-28a(+)-AgaDcat used for recombinant expression of His-tagged β-agarase AgaDcat.
